# Ubiquitin carboxy-terminal hydrolase L1 *(UCHL1) *S18Y polymorphism in Alzheimer's disease

**DOI:** 10.1186/1750-1326-5-11

**Published:** 2010-03-19

**Authors:** Madeleine Zetterberg, Annica Sjölander, Malin von Otter, Mona Seibt Palmér, Sara Landgren, Lennart Minthon, Anders Wallin, Niels Andreasen, Kaj Blennow, Henrik Zetterberg

**Affiliations:** 1 Institute of Neuroscience and Physiology, Department of Clinical Neuroscience and Rehabilitation, Section of Ophthalmology, The Sahlgrenska Academy at the University of Gothenburg, Mölndal, Sweden; 2 Institute of Neuroscience and Physiology, Department of Psychiatry and Neurochemistry, The Sahlgrenska Academy at the University of Gothenburg, Mölndal, Sweden; 3 Institute of Biomedicine, Department of Clinical Chemistry and Transfusion Medicine, The Sahlgrenska Academy at the University of Gothenburg, Göteborg, Sweden; 4 Institute of Neuroscience and Physiology, Department of Pharmacology, The Sahlgrenska Academy at the University of Gothenburg, Göteborg, Sweden; 5 Clinical Memory Research Unit, Department of Clinical Sciences, Lund University, Lund, Sweden; 6 Neuropsychiatric Clinic, Malmö University Hospital, Malmö, Sweden; 7 Memory clinic, M51, Department of Geriatric Medicine, Karolinska University Hospital, Huddinge, Stockholm, Sweden

## Abstract

Alzheimer's disease (AD) is characterized by protein aggregates, *i.e*. senile plaques and neurofibrillary tangles. The ubiquitin-proteasome system has been proposed a role in proteolytic removal of these protein aggregates. Ubiquitin carboxy-terminal hydrolase L1 (UCHL1) is a de-ubiquitinating enzyme with important functions in recycling of ubiquitin. The S18Y polymorphism of the *UCHL1 *gene confers protection against Parkinson's disease. In this study, the genotype and allele frequencies of the *UCHL1 *S18Y polymorphism were investigated in 452 AD patients and 234 control subjects, recruited from four memory clinics in Sweden. Using a binary logistic regression model including *UCHL1 *allele A and *APOE *ε4 allele positivity, age and sex as covariates with AD diagnosis as dependent variable, an adjusted OR of 0.82 ([95% CI 0.55-1.24], *P *= 0.35) was obtained for a positive *UCHL1 *allele A carrier status. The present study thus do not support a protective effect of the *UCHL1 *S18Y polymorphism against AD.

## Findings

Aggregation of aberrant proteins is a hallmark of several neurodegenerative diseases, including Alzheimer's (AD), Huntington's (HD) and Parkinson's (PD) diseases. In this context, the ubiquitin-proteasome system (UPS) has been ascribed a central role in preventing the formation of pathological protein aggregates by proteolytic removal of defect proteins [1]. Proteins destined for degradation by the UPS are labelled with a 76-amino acid peptide, ubiquitin, through a series of conjugation steps by the E1, E2 and E3 enzymes respectively. There are also two classes of de-ubiquitinating enzymes; the ubiquitin-specific processing proteases (UBPC) and the ubiquitin carboxy-terminal hydrolase family (UHC). For recent reviews on the UPS, see [2,3]. Ubiquitin carboxy-terminal hydrolase L1 (UCHL1) is an isoform of the UHC group, expressed mainly in neurons and testis/ovary. However, UCHL1 has also been found in cells of the human diffuse neuroendocrine system and is expressed is several forms of cancer [4-6]. UCHL1 has an important role in recycling of ubiquitin through hydrolysis of peptide-ubiquitin bonds and processing of ubiquitin precursors, but it also possesses ubiquitin ligase activity [7]. Moreover, it has been shown that UCHL1 associates with monoubiquitin and elongates its half-life, thus ensuring stability of ubiquitin within neurons [8].

Several genetic variants of the *UCHL1 *gene have been described; both those leading to gain-of-function and those resulting in loss-of-function. The I93 M mutant results in 50% reduced activity [9], whereas the S18Y variant exhibits increased hydrolytic activity [10]. Other studies showed comparable hydrolase activity of the *UCHL1 *S18Y variant when compared to the wild type enzyme, but a reduction in ligase activity [11]. It has also been demonstrated that the *UCHL1 *S18Y polymorphism has a specific ability to act as a potent antioxidant in neuronal cell culture systems [12].

A number of reports have demonstrated a protective effect of the S18Y polymorphism against sporadic PD in different populations [13-16], although conflicting data exist [17]. As for AD, data on *UCHL1 *genotype frequencies and its effect on risk of AD is scarce and conflicting [18,19]. The purpose of this study was to investigate the *UCHL1 *S18Y polymorphism in AD patients and controls in the Swedish population. Given that we, for subsets of the participants, have previously collected data on levels of CSF biochemical markers and neuropathological scores for AD, associations between the *UCHL1 *S18Y polymorphism and these variables could be investigated in addition to the genetic risk analysis.

The study population consisted of 452 patients with AD and 234 subjects without dementia, all of Swedish nationality. The AD patients and control subjects were recruited and diagnosed from four memory clinics in Sweden; Malmö (371 patients, 48 controls), Huddinge (52 patients, 70 controls), Göteborg/Mölndal (29 patients, 95 controls) and Linköping (21 controls). The patients were invited to participate at their first visit to the respective clinic, where they also gave their informed consent. The control subjects were recruited by advertisement in the local newspapers or at senior citizen organization meetings. A few of these controls were spouses or unrelated friends of the patients. The study was approved by the local Ethical Commissions at the respective academic center and the tenets of the Declaration of Helsinki were followed. Parts of the AD and the control groups in this work have also been included in previous studies on other polymorphisms [20,21].

All AD patients underwent clinical examination, neuropsychological evaluations including Mini-Mental State Examinaton (MMSE), computed tomography (CT) or magnetic resonance imaging (MRI) of the brain and routine blood analyses. The healthy elderly controls were classified as non-demented on the basis of a structured interview performed by a research nurse that included information on health history, lifestyle-related variables and psychosocial situation. Only controls with an MMSE score of at least 26 were included in the study. In addition, 65 years was set as the youngest age of controls for participation in the study.

Patients were clinically diagnosed with probable late-onset AD according to the NINCDS-ADRDA criteria [22] by a dementia investigation team that included specialists in geriatric medicine and psychiatry. To avoid inclusion of cases with familial Alzheimer's disease (FAD) only patients 60 years of age or older were included in the study. In addition, all patients were questioned about their family history regarding AD and those with suspect heredity were excluded. Individuals with significant psychiatric or somatic diseases other than AD were excluded. Data on CSF biomarkers, including the concentrations of total-tau (T-tau), phospho-tau_181 _(P-tau_181_) and Aβ_1-42 _was available for 260 of the AD patients and 117 of the controls. Neuropathological diagnosis and scores based on senile plaques and neurofibrillary tangles were available for 65 of the AD patients and 80 of the controls. Genomic DNA was extracted from whole blood samples and brain tissue using standard methods. *APOE *(gene map locus 19q13.2) genotyping had previously been performed by minisequencing, as described in detail [23].

The *UCHL1 *(gene map locus 4p14; [Entrez gene ID: 7345]) S18Y, 54C>A polymorphism (rs5030732) was analyzed using the Dynamic Allele Specific Amplification (DASH) tehnology as described earlier [24]. The PCR were carried out with HotStarTaq DNA Polymerase^® ^(QIAGEN, Hilden, Germany) in a final volume of 25 μl, containing 5-20 ng of template DNA. Optimal conditions were: 1 mM MgCl_2_, 0.2 mM dNTPs, 0.02 U Taq polymerase, 0.16 pmol/μl of the forward biotinylated primer (5'Biotin-GCCGCCTTGTCTCCTCTCAGCAG3') and 0.78 pmol/μl of the reverse primer (5'GTCACTGGCCTGCGACCCC3'), (Invitrogen, Paisley, UK) in 1 × PCR buffer (Roche, Mannheim, Germany). The cycling profile was: 15 min at 95°C, then 39 cycles: 30 sec at 95°C, 45 sec at 60°C, 45 sec at 72°C and finally 10 min at 72°C. To identify *UCHL1 *alleles the probe 5'GACAGAAACGCACTTGT-Rox3' (MWG Biotech, London, United Kingdom) was used. The accuracy of the DASH method was verified by DNA sequencing of 15 individuals representing the different *UCHL1 *genotypes (five of each genotype).

Primary analyses compared differences between the AD patients and control subjects regarding age, sex, MMSE score, biochemical markers, neuropathological scores, genotype and allele frequencies using Fisher's exact χ^2 ^test, *t*-test and Mann-Whitney U test. Secondary analysis of *UCHL1 *allele A-carrier status was performed with a binary logistic regression model with diagnosis (AD versus control) as dependent variable and allele positivity, age, sex and *APOE *ε4 allele status as independent variables. Significance was set at *P *< 0.05. SPSS 16.0 (SPSS Inc, Chicago, Il) was used as statistic software.

In the AD group the mean age was 76.2 (SD 6.4) years (range 60-102 years) and 299 (66.2%) were women. In the control group the mean age was 75.9 (SD 7.1) years (range 65-94 years) and 127 (58.8%) were women. A summary of demographic and clinical characteristics is presented in table 1, including MMSE score, CSF biomarkers, neuropathological score and *APOE *ε4 allele-carrier status.

**Table 1:** Demographic, clinical and genetic characteristics of patients with AD and controls

Characteristic	AD patients (n = 452)	Controls (n = 234)	*P*-value
Sex, f/m (%)	299/153 (66.2/33.8)	127/89 (58.8/41.2)	0.071*
	n = 452	n = 216	
Age, years,	76.2 ± 6.4 (60-102)	75.9 ± 7.1 (65-94)	0.215^†^
Mean ± SD (range)	n = 341	n = 216	
MMSE score, Median (25^th ^-- 75^th ^percentiles)	22 (19-25) n = 367	29 (29-30) n = 113	<0.001^‡^
T-tau (pg/ml), Mean ± SD	621 ± 319 n = 260	376 ± 162 n = 117	<0.001^†^
P-tau_181 _(pg/ml), Mean ± SD	76.9 ± 31.2 n = 260	63.3 ± 19.9 n = 91	<0.001^†^
Aβ_1-42 _(pg/ml), Mean ± SD	409 ± 100 n = 260	720 ± 200 n = 117	<0.001^†^
SPs and NFTs score, Median (25^th ^-- 75^th^percentiles)	7 (6-8.5) n = 65	1 (0-3) n = 80	<0.001^†^
*APOE ε4 *allele-carriers n (%) number of *ε4 *alleles:			
0	128 (28.3)	156 (72.2)	<0.001*
1	238 (52.7)	55 (25.5)	<0.001*
2	86 (19.0)	5 (2.3)	<0.001*
	n = 452	n = 216	

*Fisher's Exact Test, ^†^Mann-Whitney U-test. AD = Alzheimer's disease, SD = Standard Deviation, MMSE = Mini Mental State Examination, Aβ = β-amyloid, SPs = senile plaques, NFTs = neurofibrillary tangles

Genotype and allele frequencies for the *UCHL1 *S18Y polymorphism are shown in table 2. There was a significant overrepresentation of the heterozygous genotype (AC) in the control group (p = 0.03). There were no significant differences between the controls and the cases with regard to the homozygous genotypes however, even if the CC genotype was overrepresented among the AD patients with a border-line p-value (0.054). In addition, when analyzing *UCHL1 *allele A-carrier status (dominant approach) a positive allele A-carrier status was slightly overrepresented in the control group (p = 0.054). The distributions of the *UCHL1 *genotypes were in Hardy-Weinberg equilibrium for the control group (*P *= 0.602 for AA, *P *= 0.692 for AC and *P *= 0.846 for CC) as well as for the AD group (*P *= 0.436 for AA, *P *= 0.647 for AC and *P *= 0.825 for CC).

**Table 2:** *UCHL1 *genotype and allele frequencies in patients with AD and controls

*UCHL1 *genotype frequencies, n (%)	AD patients (n = 452)	Controls (n = 234)	*P*-value*
AA	15 (3.3)	6 (2.6)	0.649
AC	112 (24.8)	77 (32.9)	0.030
CC	325 (71.9)	151 (64.5)	0.054
			
***UCHL1 *allele frequencies, n (%)**^†^			
allele A	142 (15.7)	89 (19.0)	0.128
allele C	762 (84.3)	379 (81.0)	
			
***UCHL1 *allele A-carrier n (%)**	127 (28.1)	83 (35.5)	0.054
			
***UCHL1 *allele C-carrier n (%)**	437 (96.7)	228 (97.4)	0.649

*Fisher's Exact Test, ^†^There are two alleles per subject, yielding n = 904 for AD patients and n = 468 for controls. AD = Alzheimer's disease.

Using a binary logistic regression model including *UCHL1 *allele A and *APOE *ε4 allele positivity, age and sex as covariates with AD diagnosis as dependent variable, an adjusted OR of 0.82 ([95% CI 0.55-1.24], *P *= 0.35) was obtained for a positive *UCHL1 *allele A carrier status (table 3). On the basis of previously reported *UCHL1 *allele frequencies, a standardized difference of 0.29 was calculated [15]. This yielded a power of the study of 96%, indicating that the lack of association was not spurious. CSF T-tau, P-tau_181 _or Aβ_1-42 _levels were not affected by the *UCHL1 *S18Y polymorphism (Table 4). Neither did neuropathological scores of senile plaques and neurofibrillary tangles exhibit any associations with the *UCHL1 *polymorphism (Table 4). Further, we compared the *UCHL1 *allele A-positivity for AD patients and control subjects respectively between the different centers and found no differences (not shown).

**Table 3:** Binary logistic regression of AD diagnosis versus *UCHL1 *allele A- and *APOE *allele ε4-carrier status, age and gender.

Variable, n = 557	B	**S.E**.	OR	95%CI	*P-*value
Female gender	0.29	0.20	1.34	0.90-2.00	0.147
Age, years	0.01	0.02	1.01	0.98-1.04	0.456
*UCHL1*, allele A positivity	-0.20	0.21	0.82	0.55-1.24	0.348
*APOE*, allele ε4 positivity	1.96	0.20	7.09	4.83-10.4	<0.000

AD = Alzheimer's disease, B = Regression coefficient, S.E. = Standard error, OR = Adjusted odds ratio, CI = Confidence interval

**Table 4:** CSF biomarkers and neuropathological score in relation to *UCHL1 *allele A-carrier status in AD patients and control subjects

	*UCHL1 *allele A-carrier status		
CSF Biomarker	positive	negative	*P*-value*
T-tau (pg/ml), Mean ± SD			
AD patients	640 ± 368	613 ± 297	0.790
Controls	367 ± 175	381 ± 156	0.528
			
P-tau_181 _(pg/ml), Mean ± SD			
AD patients	80.6 ± 35.1	75.3 ± 29.4	0.434
Controls	58.8 ± 16.7	65.7 ± 21.1	0.159
			
Aβ_1-42 _(pg/ml), Mean ± SD			
AD patients	406 ± 91.5	410 ± 104	0.996
Controls	749 ± 195	703 ± 202	0.246
			
**SPs and NFTs score**			
Median (25^th ^-- 75^th ^percentiles)			
AD patients	7 (5-8)	7 (6-9)	0.495
Controls	2 (0-3.5)	0 (0-2)	0.122

CSF = cerebrospinal fluid, AD = Alzheimer's disease, SPs = senile plaques, NFTs = neurofibrillary tangles, Aβ = β-amyloid, *Mann-Whitney U-test

Aggregation of proteins is a major feature of several neurodegenerative disorders, including Alzheimer's disease (AD). Several lines of evidence suggest that the ubiquitin-proteasome system (UPS) is involved in the pathogenesis of AD [1]. Accumulation of ubiquitin in senile plaques and neurofibrillary tangles [25-28], changes in proteasome subunit composition in AD [29] and an association of AD with polymorphic variants of UBQLN1, encoding for ubiquilin which is a ubiquitin-like protein [30], are some of the signs pointing towards a role of the UPS in AD. Of special interest is the finding that UCHL1 is oxidized in AD and that it is down-regulated in affected brain areas of AD patients [31,32]. Also interesting is the finding that exogenous UCHL1 ameliorated β-amyloid-induced synaptic and memory dysfunction in an AD mouse model [33].

The S18Y polymorphism of the *UCHL1 *gene is associated with lower incidence of Parkinson's disease [13-16]. The mechanism for this protective effect is not known, but it may be at least partially explained by the increased antioxidative capacity demonstrated in neuronal cells expressing the *UCHL1 *S18Y variant [12]. Little is known about its effect on AD prevalence. A Chinese study has demonstrated lower frequencies of the A allele and the AA genotype in female AD patients as compared to female controls [19]. However, a genetic study in a Colombian population could not find an association between *UCHL1 *genotypes and AD [18]. The number of genome-wide association studies in the AD field has increased rapidly; none of these has reported the *UCHL1 *S18Y polymorphism among the significant SNPs found however [34,35].

The allele and genotype frequencies of the *UCHL1 *S18Y polymorphism seen in this study are in accordance with previous results in Swedish populations [13]. The present study do not support a role of the *UCHL1 *S18Y polymorphism AD however.

## Competing interests

The authors declare that they have no competing interests.

## Authors' contributions

MZ participated in the design of the study, analyzed the data statistically and drafted the manuscript. AS performed genetic analyses and helped to draft the manuscript. MvO, MSP and SL performed genetic analyses and revised the manuscript critically. LM, AW and NA collected clinical material and revised the manuscript critically. KB participated in the design of the study and revised the manuscript critically. HZ conceived of the study, helped in analyzing the data and helped in drafting the manuscript.

All authors have read and approved the final manuscript.
